# Tolosa-Hunt Syndrome With Highly Elevated Interleukin-6 Levels

**DOI:** 10.7759/cureus.113662

**Published:** 2026-07-30

**Authors:** Robert Fekete

**Affiliations:** 1 Neurology, New York Medical College, Valhalla, USA

**Keywords:** cavernous sinus, il-6, interleukin-6, ophthalmoplegic migraine, tolosa-hunt syndrome

## Abstract

Tolosa-Hunt syndrome is a rare disorder of idiopathic granulomatous orbital inflammation associated with ipsilateral orbital pain and cranial nerve palsy.

The patient is a 42-year-old Hispanic female with Ecuadorian ancestry who presented in remission of episodes of horizontal diplopia, left eye proptosis, and 8/10 sharp left eye pain that occurred 10 years ago and four years ago. Both episodes resolved with about five weeks of oral methylprednisolone dosing prescribed at onset of symptoms. She is successfully maintained on oral methylprednisolone six-day dose pack about twice yearly for moderate orbital pain. At the time of the diplopia four years ago, Magnetic Resonance Imaging showed a left cavernous sinus mass, which resolved on subsequent imaging. Her interleukin 6 (IL-6) serum level done during remission and during clinical episodes was highly elevated at 90.5 pg/ml (normal <5 pg/ml) and 68.7 pg/mL, respectively. Lower level during clinical episode may be due to use of oral steroid medication. The cytokine elevation may provide clues regarding the pathophysiology of this unusual neurological disorder.

## Introduction

Tolosa-Hunt syndrome is a granulomatous orbital inflammation that leads to unilateral orbital pain and ophthalmoplegia. There is overlap between our current understanding of the syndrome and historical descriptions of similar conditions. Charcot initially defined ophthalmoplegic migraine as "a condition associated with migraine in which there is a palsy of an ocular nerve, usually the third, temporary at first and later becoming permanent" [1]. Eduardo Tolosa first described a case of ophthalmoplegia due to paralysis of the third, fourth, and sixth cranial nerves in 1954. He found that the patient had a periarteritic lesion of the carotid siphon, with the intracavernous portion of the carotid artery surrounded by granulomatous tissue [2]. Hunt in 1961 discussed painful ophthalmoplegia as being associated with low-grade inflammation of the carotid sinus and having pain that is described as “boring or gnawing” as opposed to throbbing pain of classical migraine [3]. Under the International Classification of Headache Disorders (ICHD) (second edition), the syndrome was reclassified under cranial neuropathies but still retained the migraine name [4]. In the third edition of ICHD, it is called recurrent painful ophthalmoplegic cranial neuropathy (RPON) and requires absence of mass lesion [5]. On the other hand, Tolosa-Hunt syndrome is defined as unilateral pain associated with cranial nerve paresis caused by granulomatous inflammation in the cavernous sinus [5].

Differential diagnosis of Tolosa-Hunt syndrome includes cavernous sinus thrombosis, lymphoma, autoimmune disorders such as sarcoidosis and systemic lupus erythematosus (SLE), and infectious diseases including tuberculosis, syphilis, and Lyme disease. Workup centers on excluding these autoimmune and infectious disorders via serum and cerebrospinal fluid testing, Magnetic Resonance Imaging (MRI) of the brain to characterize the orbital mass, and vessel imaging such as Magnetic Resonance Venography (MRV) to exclude cavernous sinus thrombosis. The objective of the case report is to highlight the clinical features, diagnostic process, and treatment of this unusual headache syndrome as well as to review interleukin (IL)-6 levels in this case, as this cytokine could be a potential driver of the granulomatous inflammation in Tolosa-Hunt syndrome.

This article was previously posted to the Research Square preprint server on April 1, 2025 [6].

## Case presentation

The patient is a 42-year-old Hispanic female with Ecuadorian ancestry who presented for follow-up of mild headaches. She has a history of episodes of horizontal diplopia, left eye proptosis, and 8/10 left eye sharp pain. Episodes last for approximately six weeks.

Ten years ago, she had an episode of severe left trigeminal V1 distribution and retroorbital pain, proptosis and diplopia horizontally with sixth nerve palsy. There was no otitis media to form the triad of Gradenigo syndrome. There was no contributory past medical, social, or family history. She was initially diagnosed with ophthalmoplegic migraine [5,7]. The associated headaches were migraine-like with 8/10 sharp pain, photophobia, phonophobia, and nausea with diplopia occurring at the same time. Headache and diplopia would last for about a week. Headache would improve when she stopped activity and went to a dark room. Her examination was significant for proptosis of the left eye and partial left sixth nerve palsy. The first episode resolved after the introduction of methylprednisolone 4 mg twice daily for a month. 

The second severe episode with the same symptoms and examination findings happened four years ago. This was treated with methylprednisolone 4 mg twice a day for a month and one methylprednisolone 4 mg, six-day, 21-tablet dose pack with full resolution of symptoms.

Subsequently, she would experience up to three episodes per year of moderate 5/10 left retroorbital pain without proptosis or diplopia, treated with just one 21-tablet methylprednisolone dose pack at a time with resolution. Patient adheres to this treatment and does not experience adverse effects.

Eleven months later, the patient experienced severe 10/10 left retroorbital pain, with left proptosis and horizontal diplopia. She visited the office on day five of a six-day methylprednisolone 4 mg 21-tablet dose pack. Given still persistent symptoms, she was prescribed methylprednisolone 4 mg tabs orally daily for seven days, then one tab every other day for six days. This led to resolution of symptoms.

Initial noncontrast MRI brain 10 years ago was normal on coronal T2 fat-saturated (Figure 1) and Spectral Attenuated Inversion Recovery (SPAIR) images (Figure 2).

**Figure 1:**
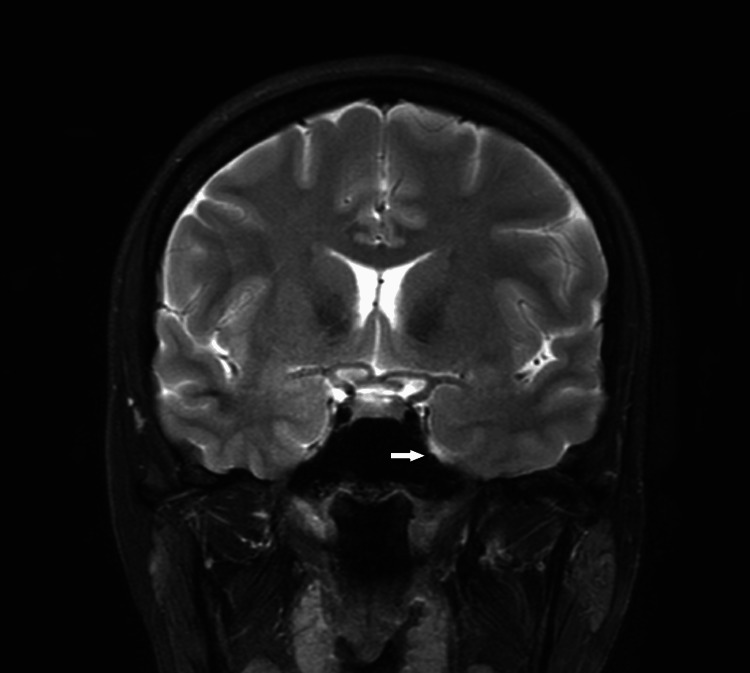
Initial normal noncontrast MRI brain coronal T2 fat-saturated image. Initial normal noncontrast MRI brain coronal T2 fat-saturated image obtained 10 years ago. There is prominence of CSF next to left temporal lobe (arrow), but cavernous sinus is normal.

**Figure 2:**
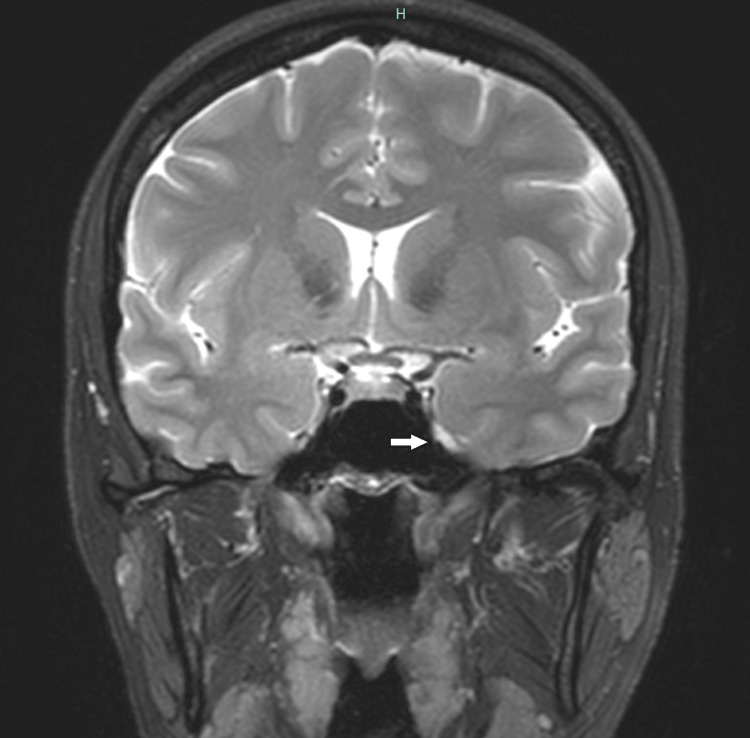
Initial normal noncontrast coronal MRI SPAIR image. Initial normal noncontrast coronal MRI Spectral Attenuated Inversion Recovery (SPAIR) image obtained 10 years ago. There is prominence of CSF next to left temporal lobe (arrow), but cavernous sinus is normal.

The patient was already treated with oral steroids for a month at the time MRI brain was performed, which could have led to the normal result.

MRI brain performed four years ago demonstrated mass-like enlargement of the left cavernous sinus on a type of 3D Fluid-Attenuated Inversion Recovery (FLAIR) MRI sequence that used compressed sensing (SHC) and spatial sensitivity encoding (SENSE) technique in the axial (Figure 3) and coronal (Figure 4) views as well as post-contrast coronal T1 views (Figure 5). 

**Figure 3:**
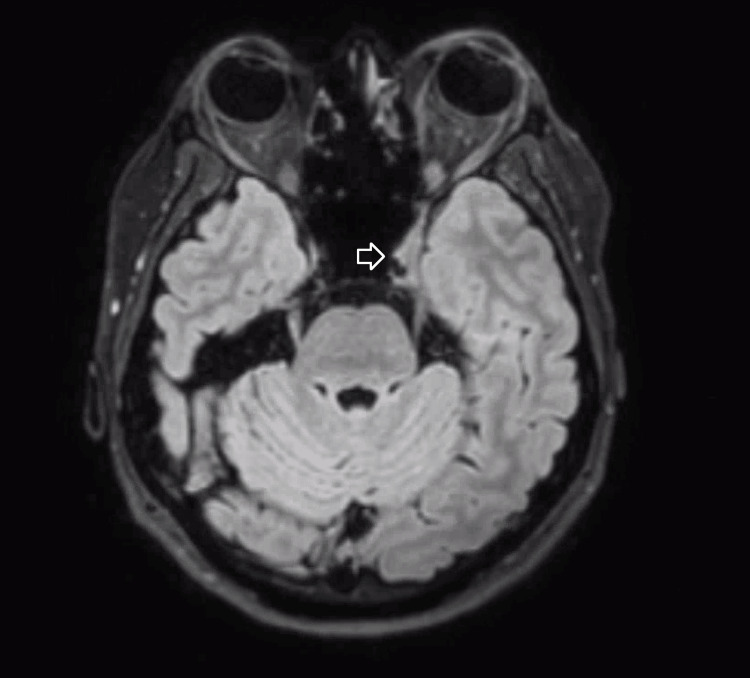
Noncontrast axial MRI image obtained four years ago with arrow showing left cavernous sinus mass.

**Figure 4:**
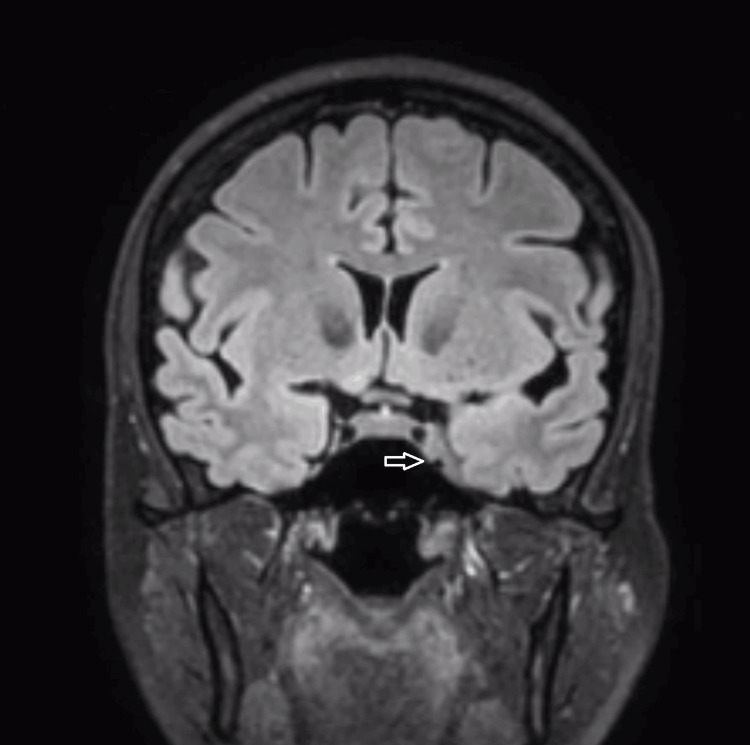
Noncontrast coronal MRI image obtained four years ago with arrow showing left cavernous sinus mass.

**Figure 5:**
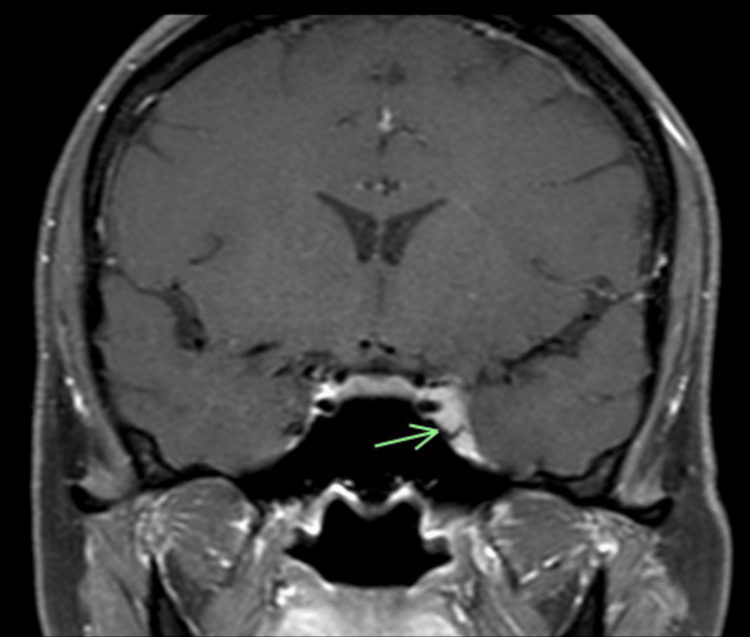
Post-contrast T1 coronal MRI image from four years ago showing enlargement of the left cavernous sinus (arrow).

The region of enhancement was defined as 18 mm by 5 mm by 17 mm in size. MRV brain was normal. Follow-up MRI brain performed eight months later showed a decreased size of the left cavernous sinus on axial T2-weighted Spectral Presaturation with Inversion Recovery (SPIR) sequence (Figure 6). T2 SPIR coronal (Figure 7) and T1 post-contrast axial imaging (Figure 8) two years ago showed resolution of the left cavernous sinus mass (Figure 7).

**Figure 6:**
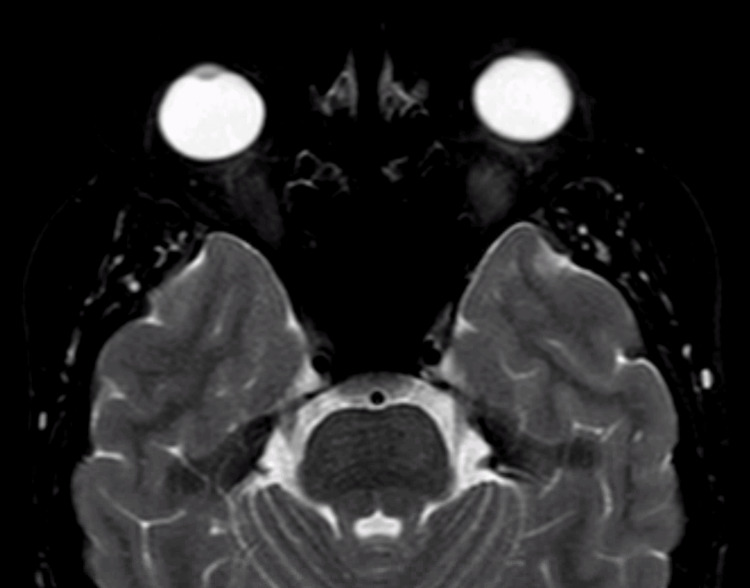
Follow-up MRI brain. Follow-up MRI brain performed eight months after imaging in Figures 1, 2 showing a decreased size of the left cavernous sinus on axial T2-weighted Spectral Presaturation with Inversion Recovery (SPIR) sequence.

**Figure 7:**
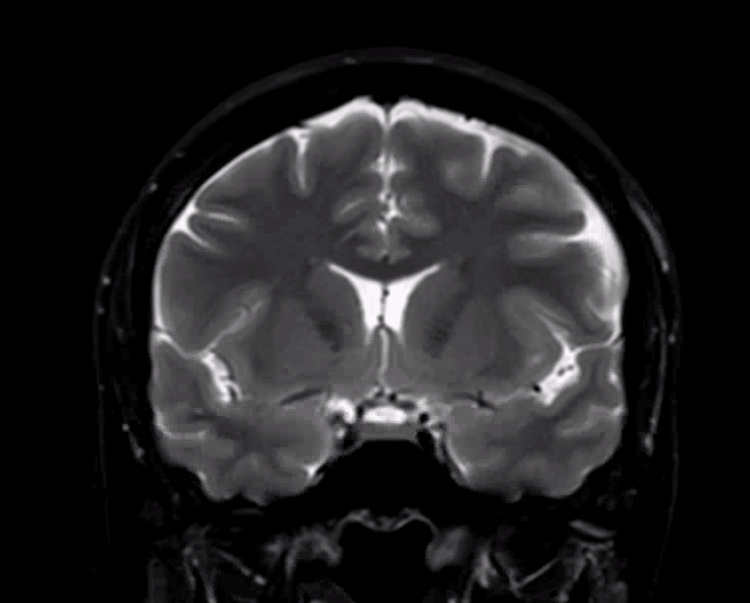
T2 Spectral Presaturation with Inversion Recovery (SPIR) MRI coronal image obtained two years ago showing resolution of the left cavernous sinus mass.

**Figure 8:**
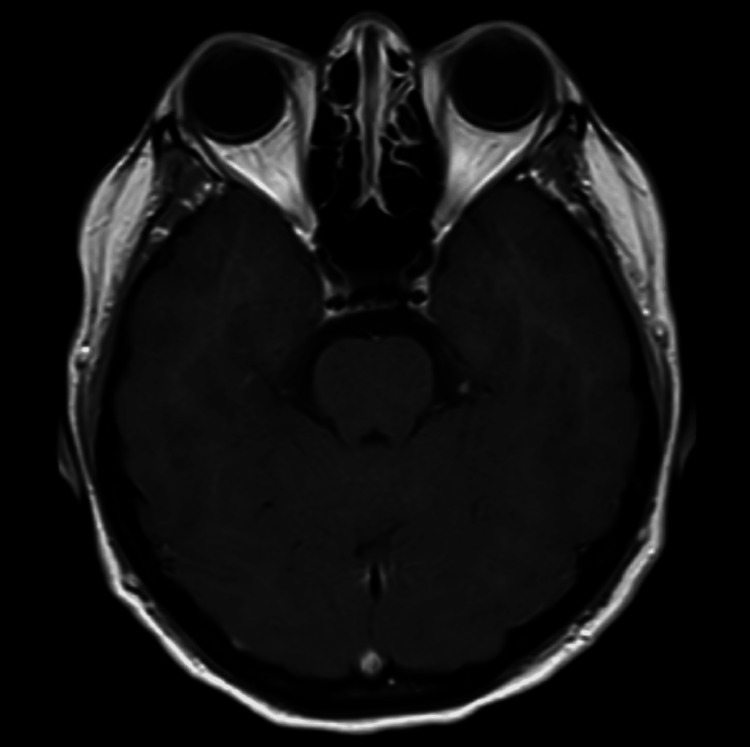
Axial T1 post-contrast image showing resolution of the left cavernous sinus mass.

MRI with eye movement analysis is not available at our institution. A timeline of her symptoms, examination findings and MRI imaging results is provided in Table 1.

**Table 1:** Timeline of symptoms, examination findings, and MRI imaging results.

Time	Symptoms	Examination Findings	MRI imaging results
t=10 years ago	Left V1 and retro orbital pain, diplopia	Left proptosis and 6^th^ nerve palsy	Normal non-contrast MRI brain
t=4 years ago	Left V1 and retro orbital pain, diplopia	Left proptosis and 6^th^ nerve palsy	Mass like enlargement of left cavernous sinus
Two years ago to t=0	Three episodes per year of moderate 5/10 left retroorbital pain without proptosis or diplopia	Normal	Normal
T=11 months	Severe episodes of 10/10 left retroorbital pain with proptosis and horizontal diplopia	Left proptosis and 6^th^ nerve palsy	Not obtained given rapid resolution of symptoms

Cerebrospinal fluid (CSF) testing during the acute episode four years ago was negative with red blood cell count 1 cell/µL, white blood cell count 0 cells/ µL, protein level of 35 mg/dL, and glucose at 65 mg/dL as well as negative Venereal Disease Research Laboratory (VDRL) test. CSF and serum anti-Borrelia antibody tests were negative.

Serum fluorescent treponemal antibody and serum protein electrophoresis were normal. HgbA1c was normal at 5.4%. Serum QuantiFERON-TB Gold Plus (QIAGEN, Hilden, Germany) and HIV tests were negative.

A year ago, she had normal serum inflammatory markers except for low C3 and low C4 in the setting of positive anti-nuclear antibody (Table 2). 

**Table 2:** Serum laboratory investigations.

Test name	Result	Reference Range
Erythrocyte sedimentation rate (ESR)	2	0-20
C-reactive protein (CRP)	<0.1 mg/dL	<0.1 mg/dL
Angiotensin converting enzyme	23 U/L	9-67 U/L
C1q	6.0 mg/dL	5-8.6 mg/dL
Anti-neutrophil cytoplasmic antibodies (ANCA)	<20	<20
Anti-double-stranded DNA (dsDNA) antibody	<12.3 IU/mL	<12.3 IU/mL
Anti-nuclear antibody (ANA)	positive, speckled, 1:80	negative
C3	65 mg/dL (low)	83-180 mg/dL
C4	8 mg/dL (low)	18-45 mg/dL

Rheumatological consultation regarding these values was appreciated with the patient not meeting the diagnostic criteria of rheumatological disorders, including SLE, in the setting of negative anti-dsDNA.

Her cytokines outside the episodes were significant for IL-10 elevated to 5.0 pg/mL (normal <=2.8) and IL-6 was elevated to 90.5 pg/mL (normal <=2.0) (Table 3). Other cytokines, IgG subtypes, and IgA subtypes were normal (Table 4). During a recent exacerbation, also with 5/5 left retroorbital pain and proptosis, she took methylprednisolone 4 mg dose pack for six days. Cytokine panel performed during day five showed elevated IL-6 to 68.7 pg/mL (Table 3). The lower level of IL-6 during the episode may have been secondary to use of oral methylprednisolone. 

**Table 3:** Cytokine levels at t=0 (remission) and t=11 months (acute episode).

Cytokine	t=0	t=11 months	Normal
Tumor Necrosis Factor alpha	<1.7 pg/ml	<1.7 pg/ml	<=7.2
Interleukin 2	<2.1 pg/ml	<2.1 pg/ml	<=2.1
Interleukin 2 Receptor, soluble	474.9 pg/ml	474.9 pg/ml	175.3-858.2 pg/ml
Interleukin 12	<1.9 pg/ml	<1.9 pg/ml	<=1.9 pg/ml
Interferon gamma	<4.2 pg/ml	<4.2 pg/ml	<=4.2 pg/ml
Interleukin 4	<2.2 pg/ml	<2.2 pg/ml	<=2.2 pg/ml
Interleukin-5	<2.1 pg/ml	<2.1 pg/ml	<=2.1 pg/ml
Interleukin-10	5.0 pg/ml	<2.8 pg/ml	<=2.8 pg/ml
Interleukin-13	2.0 pg/ml	<1.7 pg/ml	<=2.3 pg/ml
Interleukin-17	<1.4 pg/ml	<1.4 pg/ml	<=1.4 pg/ml
Interleukin-1-beta	<6.5 pg/ml	<6.5 pg/ml	<=6.7 pg/ml
Interleukin-6	90.5 pg/ml	67.8 pg/ml	<=2.0 pg/ml
Interleukin-8	<3.0 pg/ml	<3.0 pg/ml	<=3.0 pg/ml

Given MRI findings of a left cavernous sinus mass indicating orbital granulomatous inflammation ipsilateral to her headache (Figures 1, 2), she was diagnosed with Tolosa-Hunt syndrome in accordance with ICHD 2nd Edition and 3rd Edition criteria for Tolosa-Hunt syndrome [5,8]. Thorough serum and spinal fluid testing did not identify an alternative cause for her symptoms. She is currently asymptomatic except for occasional mild headaches. She takes the 21-tablet methylprednisolone dose pack twice a year on average as needed for headaches that are moderate in intensity. 

## Discussion

Tolosa-Hunt syndrome is considered to be a syndrome of idiopathic orbital inflammation. A granulomatous lesion either by biopsy or MRI visualization is required for the diagnosis [5]. The estimated annual incidence is one case per million per year [9]. There is no sex predilection [10]. Kline and Holt report no specific age group to be affected [10]. In a systematic review by Ahmed, the average age of onset is between the fourth and fifth decades of life [11]. While there is variability between case series, most cases of Tolosa-Hunt syndrome-associated pain and cranial neuropathy improve with steroids [11], but follow-up is needed as relapses may occur. Typical treatment includes initial high-dose glucocorticoids for two to four weeks followed by gradual taper. Pulsed steroid treatment was used in this case as the patient achieved complete remission between episodes. Long-term immunosuppression with alternative immunosuppressive therapy is used in situations of incomplete response to steroids or a limiting steroid side effect such as hyperglycemic episode. Examples of second-line immunosuppressive treatments include cyclosporine, azathioprine, methotrexate, mycophenolate mofetil, and infliximab. The disorder has a good prognosis, with about 10% recurrence rate [11].

In contrast to Tolosa-Hunt syndrome, the current ICHD definition of RPON requires absence of granulomatous lesion on appropriate testing modality such as MRI imaging or biopsy. This patient met ICHD 3rd Edition diagnostic criteria for Tolosa-Hunt syndrome with unilateral periorbital and orbital pain, orbital granulomatous inflammation demonstrated by MRI, horizontal diplopia due to ipsilateral sixth nerve palsy, ipsilateral (left) severe headaches that precede or develop with appearance of granulomatous inflammatory lesion, and absence of another causative ICHD-3 diagnosis [5]. According to the ICHD-3 diagnostic criteria, in addition to abducens nerve involvement, oculomotor and trochlear nerves could also be affected. Ahmed reports additional possible optic nerve and facial nerve involvement outside of ICHD-3 criteria [11]. Hence, detailed cranial nerve examination including visual acuity and funduscopic examination, especially given the differential diagnosis of cerebral venous sinus thrombosis, is important.

Tolosa-Hunt syndrome is a diagnosis of exclusion. This patient met ICHD diagnostic criteria, but it is important to differentiate from secondary inflammatory or neoplastic causes. Differential diagnostic considerations of tuberculosis, Lyme disease, diabetes mellitus, anti-neutrophil cytoplasmic antibodies (ANCA)-associated vasculitis, and CNS infectious processes were ruled out with appropriate serum and cerebrospinal fluid testing. Angiotensin-converting enzyme (ACE) level was normal. There was suspicion of SLE given decreased C3 and C4. Rheumatology consultant ruled out SLE given that anti-dsDNA antibody testing was negative. Biopsy of the mass was not performed given resolution with oral steroid treatment. Vigilance regarding mimickers such as meningioma and lymphoma is necessary. More aggressive testing including repeat CSF evaluation and consideration of surgical biopsy is recommended if symptoms do not respond promptly to steroids. Biopsy remains the gold standard of Tolosa-Hunt syndrome diagnosis.

Other differential diagnostic considerations of painful ophthalmoplegia include vascular problems such as carotid dissection, carotid aneurysm, and cerebral venous sinus thrombosis. The patient had normal MRI angiography of the head and neck and normal MR venography of the head. Central nervous system infectious causes were ruled out with normal cerebrospinal fluid testing.

Idiopathic hypertrophic cranial pachymeningitis is a rare fibroinflammatory process of the dura mater. It may present with headache and cranial neuropathy similarly to Tolosa-Hunt syndrome and should be included in the differential diagnosis. On MRI imaging of cranial pachymeningitis, there is enhancement of basal cranial dura mater with possible extension to the cavernous sinus. Basal cranial meningeal enhancement was not seen in this patient. There is controversy whether Tolosa-Hunt syndrome is a separate disorder or a focal version of idiopathic hypertrophic cranial pachymeningitis [12].

Tolosa-Hunt syndrome is often idiopathic, but it can be associated with autoimmune conditions such as Sjögren's syndrome and SLE. Given that workup was negative for other autoimmune disorders, idiopathic etiology is favored in this case. This patient had extremely elevated IL-6 during clinical remission and during a clinical attack. Use of oral methylprednisolone may have reduced the level of IL-6 during the clinical attack. IL-6 is a pleotropic cytokine central to the transition between innate and adaptive immunity and its dysregulation is implicated in many autoimmune and inflammatory disorders. Elevated IL-6 levels drive granulomatous inflammation and vascular compromise, mechanisms relevant to Tolosa-Hunt syndrome pathophysiology in autoimmune contexts. One of the prior names of IL-6 was B cell stimulatory factor-2 (BSF-2), given its ability to trigger differentiation of activated B cells into plasmablasts and antibody-producing plasma cells [13]. IL-6 can also activate T cells. Examples of IL-6 activation and its results in other disorders include autoantibody production in SLE, and elevated IL-6 levels in relapsing remitting multiple sclerosis and rheumatoid arthritis [14]. At least theoretically, a similar process involving B-cell and T-cell activation could occur in Tolosa-Hunt syndrome via IL-6. IL-6 could be a mechanistic link when occurring as a part of a broader autoimmune syndrome but its direct role in idiopathic Tolosa-Hunt syndrome remains unproven. Cytokine measurement in this case is hypothesis-generating, and single-patient cytokine measurement is insufficient to infer causality and limits generalizability, which was added to the discussion.

Increased IL-6 signaling was also proposed in development of other autoimmune disorders. Increased IL-6 and TNF-alpha signaling secondary to heart failure (HF) is proposed as the mechanism for developing atopic dermatitis in a case report of ruptured sinus of Valsalva aneurysm. Of interest, the atopic dermatitis resolved after surgical correction of the aneurysm and resolution of HF, showing another possible link between IL-6 and autoimmune disease [15].

IL-10 was elevated by a smaller magnitude and has primarily anti-inflammatory properties. IL-10 suppresses pro-inflammatory cytokines as well as chemokines and matrix metalloproteinases [16]. It was originally named “cytokine synthesis inhibitory factor” (CSIF) [17]. It also inhibits T-cell activation and proliferative responses [18]. Hence, it is less likely to be involved in triggering episodes in this patient. Cytokine profile including IL-6 was not analyzed during symptomatic episodes, which is a limitation of this case report.

The pathology of the original case of Dr. Eduardo Tolosa [2] was reviewed by Hunt et al. in 1961 [3]. The slides show a lymphocyte and plasma cell infiltrate, reduced vascularity, and proliferation of fibroblasts leading to fibrosis. There was constriction of the internal carotid artery. With reclassification of formerly idiopathic fibroinflammatory syndromes such as Mikulicz disease as IgG4-related disease (IgG4-RD), it is of interest that this pathological finding bears shares some histopathological features with IgG4-RD diagnostic criteria, except that the layering of fibrosis is linear as opposed to the spoke wheel (storiform) type of fibrosis in IgG4-RD [19]. Storiform fibrosis is one of the pathological diagnostic hallmarks of IgG4-RD. Specific immunostaining for IgG4 was not available at the time of the historical case. The presence of fibrosis in the historical case could be a reason for incomplete recovery due to residual cranial nerve dysfunction reported in some patients. Unlike in most cases of IgG4-RD, serum testing of the current patient revealed normal IgG subsets, soluble IL-2 receptor subunit alpha level, and transforming growth factor beta-1 level (Table 4).

**Table 4:** IgG subclasses, IgA subclasses, and human transforming growth factor beta-1 levels at t=0

Test	Patient values	Normal values
Total IgG	1206 mg/dl	767-1590 mg/dl
IgG 1	626 mg/dl	341-894 mg/dl
IgG 2	487 mg/dl	171-632 mg/dl
IgG 3	85.4 mg/dl	18.4-106.0 mg/dl
IgG 4	48.6 mg/dl	2.4-121.0 mg/dl
IgA	321 mg/dl	85-499 mg/dl
IgA1	261 mg/dl	76-328 mg/dl
IgA2	68.8 mg/dl	6.9-114.3 mg/dl
Transforming Growth Factor beta-1	12123 pg/mL	<22062 pg/ml

Magnetic resonance angiography (MRA) of the head and neck were normal in the current patient.

While the current patient has not developed complications from long-term steroid use, it is necessary to monitor for adverse events from long-term steroid use such as decreased bone mineral density, hyperglycemia, hypertension, hyperlipidemia, cataracts, and adrenal suppression. The timing of cytokine level analysis during remission as opposed to during acute episode limits interpretation of these results.

While Tolosa-Hunt syndrome does resolve clinically and radiologically after oral steroid administration, the presence of fibrosis in Dr. Tolosa’s case raises the possibility of cumulative pathology. Prompt administration of steroids would be helpful in reducing the risk of fibrosis. Screening MRA, given constriction of the internal carotid artery in Dr. Tolosa’s case report, may be considered.

## Conclusions

Tolosa-Hunt is a syndrome of idiopathic granulomatous orbital inflammation which requires careful attention by the clinician to rule out other autoimmune, vascular, and neoplastic disorders. This rare syndrome can be fully treated with short courses of oral steroids. As in this case, the granulomatous orbital inflammation can fully resolve. Elevation of IL-6 may provide hypothesis-generating clues regarding the autoimmune basis of this disorder as this cytokine may be the driver of granulomatous inflammation. Single-patient cytokine measurement is insufficient to infer causality and limits generalizability.
